# HIV post‐exposure prophylaxis in community settings and by lay health workers or through task sharing: a systematic review of effectiveness, case studies, values and preferences, and costs

**DOI:** 10.1002/jia2.26448

**Published:** 2025-05-27

**Authors:** Caitlin E. Kennedy, Rahel Dawit, Ping Teresa Yeh, Michelle Rodolph, Nathan Ford, Heather‐Marie A. Schmidt, Robin Schaefer, Rachel Baggaley, Virginia Macdonald

**Affiliations:** ^1^ Department of International Health Johns Hopkins Bloomberg School of Public Health Baltimore Maryland USA; ^2^ Department of Epidemiology Johns Hopkins Bloomberg School of Public Health Baltimore Maryland USA; ^3^ Department of Global HIV Hepatitis and Sexually Transmitted Infections Programmes World Health Organization Geneva Switzerland; ^4^ UNAIDS Geneva Switzerland; ^5^ Forum for Collaborative Research University of California Berkeley California USA

**Keywords:** community delivery, HIV, lay health workers, PEP, post‐exposure prophylaxis, systematic review

## Abstract

**Introduction:**

Post‐exposure prophylaxis (PEP) for HIV prevention has been inadequately promoted, provided and used. Expanded access and task sharing could increase the HIV prevention impact of PEP, but scientific evidence to inform programmatic and policy decisions has not been synthesized.

**Methods:**

To inform World Health Organization guidelines, we conducted a systematic review of studies examining the provision of PEP in community settings, and by trained lay health workers or through task sharing. We searched CINAHL, PsycINFO, PubMed, EMBASE and scientific conferences for studies published between January 2012 and October 2023. We screened abstracts and extracted data in duplicate. The effectiveness review included randomized controlled trials and comparative observational studies; risk of bias was assessed using Cochrane Collaboration and Evidence Project tools, and the certainty of the evidence was assessed using GRADE. We also summarized implementation case studies, values and preferences studies, and cost and cost‐effectiveness studies.

**Results:**

For provision of PEP in community settings, we identified one effectiveness study, three case studies, one values and preferences study, and one cost study. Very low certainty evidence from one study in Kenya and Uganda suggested that PEP uptake, when offered as part of a dynamic prevention package, was highest in the community setting (vs. outpatient or antenatal care settings). For provision of PEP by trained lay health workers or task sharing, we identified three effectiveness studies, two case studies, four values and preferences studies, and one cost study. Very low certainty evidence from Kenya, Uganda and the United States suggested that engagement of lay providers or pharmacists increased PEP uptake and completion and decreased HIV acquisition. Studies from six countries found most health workers supported PEP provision by non‐specialist providers. One modelling study suggested community‐based provision may be cost‐effective or cost‐saving in Africa.

**Discussion:**

Evidence on expanding PEP access through community delivery or task sharing is limited but generally suggests positive outcomes, feasibility, acceptability and cost‐effectiveness of these approaches. Indirect evidence from HIV treatment and pre‐exposure prophylaxis further supports these approaches.

**Conclusions:**

Programmes should be expanded to include community delivery and task sharing to dispense, distribute, provide and monitor PEP.

## 1 INTRODUCTION

The use of antiretroviral drugs as post‐exposure prophylaxis (PEP) for HIV prevention has been shown to be effective [1] and recommended by the World Health Organization [2] for over three decades, with updates to reflect newer drug combinations, yet PEP remains an underutilized HIV prevention tool. In many parts of the world, PEP is not widely available outside of hospital settings for healthcare‐associated occupational or sexual assault exposures. Experience with pre‐exposure prophylaxis (PrEP) suggests that access in community settings and engaging a range of providers can expand access and use [3]. PEP may also have the potential to be more strategically used for HIV prevention through expanded access in community settings, and through task sharing between health workers, including trained lay health workers. In the United States, laws have been recently changed to allow pharmacists to initiate or prescribe PEP in New York (as of 2017) [4] and California (as of 2019) [5], along with several other states. Similarly, pharmacies have been proposed as a promising location for PEP prescribing to increase access in South Africa [6]. However, the scientific evidence to inform programmatic and policy decisions about expanded PEP provision has not been synthesized.

We sought to conduct a systematic review of studies related to the provision of HIV PEP in community settings, and by trained lay health workers or through task sharing.

## 2 METHODS

We reviewed effectiveness, case studies, values and preferences, and cost data related to PEP in community settings, and by trained lay health workers or through task sharing. We present methods and results for each of these separately, starting with studies of intervention effectiveness.

### 2.1 Effectiveness review

The effectiveness review covered two complementary interventions framed using the PICO (population, intervention, comparator, outcomes) approach. The first PICO question asked, should PEP be offered in community settings? The second PICO question asked, should PEP be offered by lay health workers or through task sharing?

#### 2.1.1 PICO 1: PEP in community settings

Population: Individuals eligible for PEP

Intervention: Availability of PEP in community settings (defined as non‐healthcare settings; this could include offering of PEP by community‐based organizations, community health workers based outside of facilities, mobile units or pharmacies, among other settings)

Comparator: No availability of PEP, or availability only in healthcare settings

Outcomes:

1. Quality of PEP services offered (e.g. adherence to country or international guidelines)
2. Uptake of PEP
3. Timeliness of PEP uptake (time since exposure)
4. Completion of PEP
5. HIV acquisition
6. Linkage to or uptake of appropriate additional services (e.g. PrEP, antiretroviral treatment [ART])
7. Adverse events (e.g. coercion, intimate partner violence, self‐harm, psychosocial issues, stigma/discrimination)

#### 2.1.2 PICO 2: PEP offered by lay health workers or through task sharing

### 2.2 Population: Individuals eligible for PEP

Intervention: PEP provided by trained lay health workers, as defined by the WHO as “any health worker who performs functions related to health‐care delivery; was trained in some way in the context of the intervention; but has received no formal professional or paraprofessional certificate or tertiary education degree” [5]. We also included studies that focused on task sharing, where PEP was provided by a lower‐level provider.

Comparator: PEP provided by trained health workers/higher‐level health workers or no provision of PEP

Outcomes:

1. Quality of PEP services offered (e.g. adherence to country or international guidelines)
2. Uptake of PEP
3. Timeliness of PEP uptake (time since exposure)
4. Completion of PEP
5. HIV acquisition
6. Linkage to or uptake of appropriate additional services (e.g. PrEP, ART)
7. Adverse events (e.g. coercion, intimate partner violence, self‐harm, psychosocial issues, stigma/discrimination)

Although these PICO questions focused on PEP in community settings and PEP offered by lay health workers, we also included studies comparing different settings where PEP is available (e.g. hospital vs. primary healthcare, hospital vs. pharmacy, etc.) as well as studies that compared PEP offered by different cadres of health workers (e.g. physicians vs. pharmacists, pharmacists vs. nurses, etc.), as our goal was to understand questions around decentralizing services and providing new models of care in community settings.

Studies were included in the effectiveness review if they met the following criteria:

1. Study population included individuals eligible for PEP (according to country or international guidelines).
2. Study design was a randomized trial or comparative observational study (including non‐randomized quasi‐experimental studies) that compared people who received the intervention described in the PICO question to those who received an intervention described in the PICO comparison group.
3. Measured one or more of the outcomes of interest.
4. Published in a peer‐reviewed journal or as a conference abstract between 1 January 2012 (the year when antiretroviral drugs for treatment were recommended in community settings by WHO [2]) and 16 October 2023 (database search date).

If studies combined both PEP and PrEP, or combined both PEP and HIV treatment for the purposes of assessing “biomedical HIV prevention” more generally, we reported only PEP‐specific findings if findings were disaggregated; if findings were not disaggregated, we reported findings but made clear that these were for combined interventions. Studies from any geographic region and any language were included. For studies published in languages other than English, we reviewed the English‐language abstract if one was available, or a Google translate version of the abstract if it was in a language not spoken by the study team.

### 2.3 Case studies review

Studies were included in the case studies review if they presented primary data examining the implementation of a programme for community‐based PEP or provision of PEP by trained lay health workers/task‐sharing and provided information on implementation characteristics or outcomes from this programme, but that data did not meet the pre/post or multi‐arm criteria for the effectiveness reviews. These studies could be qualitative or quantitative in nature but had to present primary data collection—think pieces and review articles were not included. These could include studies that examined barriers and facilitators to PEP in community settings or by lay health workers/task‐sharing, or studies that provided non‐comparative outcome data on the PICO outcomes listed above.

### 2.4 Values and preferences review

Studies were included in the values and preferences review if they presented primary data (qualitative or quantitative) examining the values and preferences or acceptability of community‐based PEP or provision of PEP by trained lay health workers/task‐sharing to potential beneficiaries, communities, health workers and other stakeholders. This literature could include studies examining the acceptability of various intervention options covered in the PICO questions above and service delivery preferences, among others. Studies that reported only uptake of PEP or awareness of PEP as a proxy for preferences were not included.

### 2.5 Cost review

Studies were included in the cost review if they presented primary data comparing costing, cost‐effectiveness, cost‐utility or cost‐benefit of community‐based PEP or provision of PEP by trained lay health workers/task‐sharing. Costs could include health sector costs, other sector costs, client/family costs or productivity impacts.

#### 2.5.1 Search strategy

We used a single search strategy to identify articles using terms for “PEP” and “HIV.” This broad search was intended to maximize sensitivity. We searched four online databases (CINAHL, PsycINFO, PubMed and EMBASE) for relevant peer‐reviewed publications using the following search terms:

**Pubmed**: (HIV[Title/Abstract] or HIV[MeSH]) AND (post‐exposure prophylaxis [MeSH] OR “postexposure prophylaxis”[Title/Abstract] OR “post‐exposure prophylaxis”[Title/Abstract] OR “post exposure prophylaxis”[Title/Abstract] OR PEP[Title/Abstract]))

**CINAHL**: (MH HIV OR AB HIV OR TI HIV) AND (MH “post‐exposure prophylaxis” OR AB “postexposure prophylaxis” OR AB “post‐exposure prophylaxis” OR AB “post exposure prophylaxis” OR AB PEP OR TI “postexposure prophylaxis” OR TI “post‐exposure prophylaxis” OR TI “post exposure prophylaxis” OR TI PEP)

**PsycINFO**: (MH HIV OR AB HIV OR TI HIV) AND (MH “post‐exposure prophylaxis” OR AB “postexposure prophylaxis” OR AB “post‐exposure prophylaxis” OR AB “post exposure prophylaxis” OR AB PEP OR TI “postexposure prophylaxis” OR TI “post‐exposure prophylaxis” OR TI “post exposure prophylaxis” OR TI PEP)

**EMBASE**: ((‘postexposure prophylaxis’:ab, ti OR ‘post‐exposure prophylaxis’:ab, ti OR ‘post exposure prophylaxis’:ab, ti OR pep:ab, ti) AND hiv:ab, ti)

We also searched conference abstracts from the Conference on Retroviruses and Opportunistic Infections (CROI), the International AIDS Society Conference on HIV Science (IAS) and the International AIDS Conference (IAC). We reviewed the reference lists of several previously conducted reviews [8, 9, 10] and of all included studies. Finally, we asked selected experts to propose potentially relevant studies.

#### 2.5.2 Screening process

Titles, abstracts, citation information and descriptor terms of citations identified through the search strategy were screened by a member of the review team (PTY, RD, CK). Full‐text articles were obtained for all selected abstracts and two independent reviewers (CK, RD) assessed all full‐text articles for eligibility to determine final study selection. Differences were resolved through consensus.

#### 2.5.3 Data extraction, management and analysis

Data were extracted independently by two reviewers (CK, RD) using standardized data extraction forms in Excel. Differences in data extraction were resolved through consensus. From each study, we gathered information on citation information (author, year, title, journal, language of article), location, study population, sample size, study design, intervention summary, comparator (when applicable) and study outcomes. For the effectiveness review, risk of bias at the study level was assessed using the Cochrane Collaboration risk of bias tool for randomized trials [11] and the Evidence Project risk of bias tool [12] for comparative observational studies. We also assessed risk of bias at the level of individual outcomes and assessed the overall certainty of the evidence using GRADE [13].

Data were summarized descriptively for each component of the review (effectiveness, case studies, values and preferences, and cost). For the effectiveness review, we planned to conduct a meta‐analysis using random‐effects models, but did not have enough comparable studies to combine.

## 3 RESULTS

**Figure 1:**
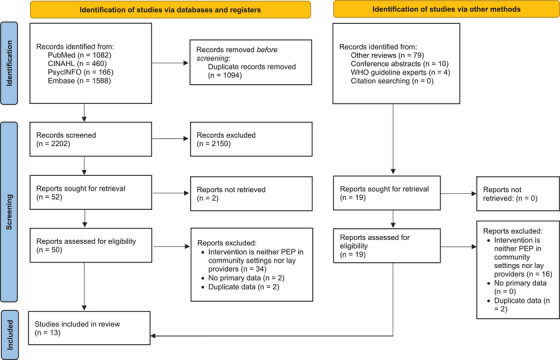
PRISMA flowchart. Disposition of articles through the search and screening process.

Figure 1 presents a PRISMA diagram showing the disposition of citations through the search and screening process [14]. Of 2202 unique citations identified through the search process, 13 studies met our inclusion criteria: three effectiveness studies, five case studies, five values and preferences studies (one of which was reported in the same abstract as a case study) and two cost studies (one of which was reported in the same abstract as an effectiveness study).

### 3.1 Effectiveness review

One study met the inclusion criteria for the effectiveness review of PEP offered in community settings (Table 1) [15]. The Sustainable East Africa Research in Community Health (SEARCH) SAPPHIRE study, conducted in Kenya and Uganda, offered PEP as part of a “dynamic choice” model of HIV prevention options, which also included PrEP and condoms. The dynamic choice model was delivered in three different settings: in antenatal care (ANC) settings and outpatient departments (OPDs), where PEP was offered by clinical officers and nurses, and in community settings, where PEP was offered by community health workers who facilitated the intervention by clinical officers from the local health centre. “Dynamic choice” refers to people being able to choose and switch between interventions, service locations and service providers. At intervention visits during weeks 4, 12 and 24, participants were asked to select a choice of HIV prevention option (PrEP, PEP, condoms only and no selection), HIV testing modality (oral self‐test or clinician‐administered rapid antibody) and preferred location for next visit (clinic vs. out‐of‐facility). At week 24, PEP use and HIV risk (report of sexual partners with HIV or unknown status and/or self‐identification as being at risk) for each of the prior six calendar months were assessed via a structured survey. There was risk of bias when comparing across settings, as populations accessing the intervention through community, ANC and OPD settings were substantially different on demographic factors (e.g. gender, age, pregnancy status). This single observational study was judged as providing very low certainty evidence for the PICO question (Supplementary Appendix). The study measured the outcome of uptake of PEP over 24 weeks of follow‐up and found that the initial choice of PEP for HIV prevention was highest in the community setting (46%) compared to the OPD and ANC settings (9% and 1%, respectively). Selection of PEP over the follow‐up study visits remained highest in the community setting over time (23% at week 24); in the ANC and OPD settings, only 3% and 11%, respectively, ever selected PEP.

**Table 1:** Description of studies included in the effectiveness review

Study	Country	PEP setting/intervention	Population	Study design	Sample size
**PICO 1: PEP offered in community settings**					
Kabami et al., 2022	Kenya and Uganda	PEP offered by CHWs in community settings as part of a dynamic HIV prevention choice model settings	Community	Non‐randomized trial	612 community participants
**PICO 2: PEP offered by lay health workers/task‐sharing**					
Kabami et al., 2022	Described above				
Grossman et al., 2020	USA	Pharmacist prescribing PEP in an infectious disease clinic	Individuals referred from an emergency department for non‐occupational exposure	Retrospective chart review before/after intervention	24 PEP clients
Lowrey et al., 2020	USA	Pharmacist prescribing free PEP, providing education and conducting a follow‐up call	Sexual assault survivors presenting to an emergency department	Retrospective chart review before/after intervention	369 PEP clients

Abbreviations: CHWs, community health workers; PEP, post‐exposure prophylaxis.

The SEARCH SAPPHIRE study was also considered to meet the inclusion criteria for the effectiveness review of PEP offered by lay health workers, along with two additional conference abstracts, both reporting on studies conducted in the United States (Table 1).

The first additional study conducted a retrospective chart review of PEP users before and after a programme which allowed a pharmacist in an infectious disease clinic to prescribe PEP following a referral from an emergency department; previously, PEP cases were seen in the infectious disease clinic without pharmacist involvement [16]. This observational study had a small sample size (*n* = 24 PEP users across both arms) and was judged as providing very low certainty evidence for the PICO question (Supplementary Appendix). This study measured two primary outcomes: PEP uptake and PEP completion. After the intervention, 16/16 (100%) of eligible clients left the clinic with PEP, compared with 5/8 (62.5%) prior to the intervention. After the intervention, 42% of those who left the clinic with PEP completed the entire PEP course and came to a follow‐up appointment, compared to 32% before the intervention (numbers not reported).

The second additional study also conducted a retrospective chart review comparing before and after implementation of a programme that involved pharmacists dispensing free PEP, providing patient education prior to discharge and conducting a follow‐up phone call after 3 months for sexual assault survivors in an emergency department [17]. The study measured the outcomes of PEP completion and HIV acquisition, but had a small number of events, and was judged as providing very low certainty evidence for the PICO question (Supplementary Appendix). PEP completion was 19.8% (*n* = 55) with the pharmacist‐delivered interventions compared with 4.3% (*n* = 4) before the intervention (the total number of charts reviewed was 369, but the sample size before and after the intervention was not reported). There were two documented cases of HIV seroconversion before the intervention and none afterwards.

No studies included in the effectiveness review measured the other PICO outcomes: quality of PEP services offered, timeliness of PEP uptake, linkage to or uptake of appropriate additional services or adverse events.

### 3.2 Case studies review

Three studies were included in the case studies review of PEP offered in community settings (Table 2). The first study trained health workers from government clinics in Kenya and Uganda on PEP with an option for those trained to offer out‐of‐facility, community‐based medication delivery [18]. Among 124 clients who sought PEP through these clinics, 85% completed PEP and no serious adverse events or HIV seroconversions were reported. Overall, 12% of visits were conducted at out‐of‐facility community‐based sites; 35% of participants had at least one out‐of‐facility visit. The second study examined police initiation of PEP at police stations for sexual assault survivors in Zambia [19]. Of 207 cases of sexual assault, about half were eligible for PEP (*n* = 104), but only 25% of these (*n* = 26) were initiated on PEP by the police. The authors noted that less than half of eligible cases (*n* = 49) presented during official police working hours, and 33% of eligible survivors who reported during official working hours received PEP, compared to 18% of those who reported on nights or weekends. No adverse events were reported. The third study examined a web‐based platform for delivering PEP in China [20]. Of 539 PEP users, nearly all (99%) started PEP within 72 hours of exposure and there were no HIV seroconversions reported.

**Table 2:** Description of studies included in the case studies review

Study	Country	PEP setting/intervention	Population	Sample size	Results
**PICO 1: PEP offered in community settings**					
Ayieko et al., 2021	Kenya and Uganda	Community‐based delivery	General population	124 PEP initiations	‐ 124 persons sought PEP; 85% completed PEP, and there were no HIV seroconversions ‐ 12% of all visits were conducted at out‐of‐facility community‐based sites; 35% of participants had ≥1 out‐of‐facility visit ‐ No serious adverse events were reported
Zama et al., 2015	Zambia	Police stations	Sexual assault survivors	207 cases of sexual assault	‐ About half of cases were PEP‐eligible, and 25% of these were initiated on PEP by the police ‐ Less than half of eligible cases presented during working hours, but eligible cases were more likely to receive PEP if during working hours (33%) than if during non‐working hours (18%)
Shan et al., 2023	China	Internet‐based service	Mostly men who have sex with men	539 PEP users	Of 539 PEP users who responded to the survey, nearly all (99%) started PEP within 72 hours of exposure and there were no HIV seroconversions reported
**PICO 2: PEP offered by lay health workers/task‐sharing**					
Roche et al., 2023	Kenya	Private pharmacies	General population	989 clients	173 clients were initiated on PEP, and 18% (32/173) of these transitioned to PrEP upon PEP completion
Mensforth et al., 2018	UK	Nurse‐delivered clinics	Not reported	27 PEP assessments	‐ Of 19 PEP prescribing decisions, 18 met local prescribing criteria ‐ Nurse prescribing was described as “comparable, if not better than” doctor prescribing

Abbreviations: PEP, post‐exposure prophylaxis; PrEP, pre‐exposure prophylaxis.

Two studies were included in the case studies review of PEP offered by lay health workers/task‐sharing (Table 2). The first study evaluated PEP delivery by pharmacists in 12 private pharmacies in Kenya [21]. Of 989 clients screened over a 6‐month period for PEP, PrEP or sexually transmitted infection (STI) testing, 173 clients were initiated on PEP, and 18% (32/173) of these transitioned to PrEP upon PEP completion. The second study trained 14 nurse non‐medical prescribers to offer PEP in nurse‐delivered clinics in the UK, compared to the usual offer through a central sexual health hub clinic and emergency department, and compared their outcomes with a sample of PEP prescribed by doctors over the same period [22]. In the 6 months after training, 27 PEP assessments were completed by nine nurses across six satellite clinics. Of 27 patient assessments, 19 received PEP, with 18/19 of those prescribing decisions meeting local prescribing criteria.

### 3.3 Values and preferences review

One study was identified for the values and preferences review of PEP offered in community settings (Table 3). This study was a cross‐sectional survey of 342 sexual and gender minorities visiting collective sex venues in New York City, USA [23]. In open‐text survey responses, participants expressed interest in such venues providing a range of free HIV and STI prevention services, including PEP. Although results were not separated for PEP services, participants felt services could be delivered in an acceptable way, although potential barriers included privacy concerns, access to health services in other locations (and thus limited perceived need for community‐based services) and negative reactions to the presence of service providers at sex venues.

**Table 3:** Description of studies included in the values and preferences review

Study	Country	Participants	Study design	Sample size
**PICO 1: PEP offered in community settings**				
Cai et al., 2023	USA	Sexual and gender minorities	Cross‐sectional survey	342
**PICO 2: PEP offered by lay health workers/task‐sharing**				
Clifford‐Rashotte et al., 2018	Canada	Nurses	Cross‐sectional survey	214
Beanland et al., 2015	Multi‐country (South Africa, USA, Lesotho, Armenia and Kenya)	Health workers	Cross‐sectional survey	306
Bellman et al., 2022	USA	Pharmacists	Semi‐structured qualitative interviews	7
Roche et al., 2023	Kenya	PEP clients and pharmacy providers	Cross‐sectional survey	Not reported

Abbreviations: PEP, post‐exposure prophylaxis; PrEP, pre‐exposure prophylaxis; STI, sexually transmitted infection; USA, United States of America.

Four studies were identified for the values and preferences review of PEP offered by lay health workers/task‐sharing (Table 3). Two of these were online, cross‐sectional surveys of non‐randomly selected PEP providers. The first study used an online survey of 214 nurses in Ontario, Canada to assess perspectives on allowing nurses to dispense PEP. Overall, 76.9% of participants indicated they would be supportive of nurse‐led PEP under medical directives [24]. The second study was a multi‐country, mixed‐methods study to examine values and preferences around PEP to inform prior WHO guidelines [25]. The online survey component was completed by 306 participants from five countries: South Africa (*n* = 90), the United States (*n* = 51), Lesotho (*n* = 16), Armenia (*n* = 16) and Kenya (*n* = 15). Of these providers, 65.5% (*n* = 110) disagreed that 28‐day prescribing should only be prescribed by HIV specialists, and 74.1% (*n* = 126) agreed that they could allow non‐HIV specialists to start PEP safely. The third study reported findings from semi‐structured qualitative interviews with staff at PEP‐prescribing pharmacies in the San Francisco Bay area, USA [26]. Of seven interview participants, all felt the California state bill that allowed pharmacists to dispense PEP was a valuable expansion of services. Finally, the fourth study, also included in the case study review, evaluated a model of PEP delivery (along with PrEP and HIV testing) in private pharmacies in Kenya [21]. Although PEP was not separated from PrEP in the analysis, acceptability was generally high. The majority (70−100%) of clients and providers reported that they liked getting/delivering PrEP/PEP at the pharmacy and that getting/delivering PrEP/PEP at the pharmacy was not hard.

### 3.4 Cost review

Two studies were included in the cost review: one for PEP offered in community settings, and one for PEP offered by lay health workers/task‐sharing (Table 4). The first study used mathematical modelling to examine a range of scenarios around wider PEP availability in communities in West, East, Central and Southern Africa [27]. This study estimated a cost of US$16.20 for 3 months of PEP availability, including a 20% additional supply chain cost to cover distribution. In the mathematical models, overall costs were lower with community PEP than with no community PEP in 92% of setting scenarios, with $18.0 million (14% of the overall HIV budget of US$127.8 million per year) savings per year over 50 years as a result of fewer people requiring ART and lower ART‐related clinic visits over the long term. Models suggested that community PEP was cost‐effective in 90% of setting scenarios and cost‐saving (with disability‐adjusted life‐years averted) in 58% of scenarios. When only examining setting scenarios in which there was a lower uptake of community PEP, it was found to be cost‐effective in 92% of setting scenarios. The second study, also included in the effectiveness review, assessed cost savings associated with a new PEP programme offered by pharmacists in the United States, which included coordinated benefits investigation and low or no‐cost medication access [16]. Through this combination of interventions, clients’ average out‐of‐pocket costs for one course of PEP ranged from US$2.25−$7.30 after the pharmacist intervention, compared to US$475.00−$3733.40 before the intervention.

**Table 4:** Description of studies included in the costs review

Study	Country	Setting	Population	Sample size
**PICO 1: PEP offered in community settings**				
Phillips et al., 2023	Multiple countries in Africa	Community‐based availability of PEP	General population	Modelling study
**PICO 2: PEP offered by lay health workers/task‐sharing**				
Grossman et al., 2020	USA	Pharmacists prescribing PEP and facilitating counselling and low or no cost medication access	Patients referred from an emergency department to an infectious disease clinic	24

Abbreviation: PEP, post‐exposure prophylaxis.

## 4 DISCUSSION

This systematic review found that research on expanding PEP access through community delivery or task sharing is limited, but existing research generally suggests positive outcomes, as well as the feasibility and acceptability of these approaches. Currently, many countries lack detailed national policy guidance on PEP [28], and PEP is not widely available outside of hospital settings for healthcare‐associated occupational or sexual assault exposures. A previously published systematic review reported that just 14% of eligible people refused PEP [29]. Expanded PEP access could increase coverage of this effective HIV prevention strategy, which remains urgently needed in a world where approximately 1.3 million people acquired HIV in 2023 [30].

While we were interested in PEP delivery by lay health workers, many of the included studies reflected more of a task‐sharing approach, where PEP services were provided by pharmacists or other health workers. While there is substantial support for community pharmacist provision of PEP [4], future research with lay health workers, including peers, would be valuable.

We did not include studies examining the PEP in pocket (“PIP”) approach, where clients are given a prescription for HIV PEP to self‐initiate in case of future high‐risk exposures. This approach has generally been offered by trained health workers in non‐community settings, so it did not meet our inclusion criteria; however, evidence over many decades suggests it may also hold promise as an additional strategy for widening appropriate access to PEP [31, 32, 33, 34, 35, 36]. Indeed, while not offered by trained health workers, the SEARCH trial presented here could be considered an example of a PEP in pocket approach. Studies of PEP in pocket have found it to be feasible and effective [31, 32, 33, 35], with individuals appropriately determining when to use PEP [31], and with few [35] to no [31, 32, 33] observed HIV seroconversions.

Community‐based delivery and task sharing have been successfully used for a range of other HIV services, providing indirect evidence that these strategies should also work for PEP. WHO supports community‐based and pharmacy‐based delivery of PrEP [3]. For HIV treatment, WHO recommends that trained non‐physician clinicians, midwives and nurses can initiate first‐line ART, trained and supervised community health workers can dispense ART between regular clinical visits and trained and supervised lay healthcare providers can distribute ART [36]. Both ART and PrEP are more complex to deliver than PEP and require longer‐term engagement with the health system. Community‐based services and task sharing have also been used for HIV testing, viral hepatitis testing and treatment, harm reduction, contraception and a range of other health services. It is reasonable to assume that provision of PEP through the same strategies would similarly result in improved access and outcomes.

WHO also recommends that HIV self‐testing may be used to deliver pre‐ and post‐exposure prophylaxis, including for initiation, re‐initiation and continuation for PrEP and initiation and follow‐up for PEP [3]. Self‐testing could facilitate community delivery and benefit PEP clients, supporting earlier access to PEP in community settings and reducing opportunity costs for clients who would not need to routinely visit health services for follow‐up after completion of a course of PEP.

This review has several limitations. Although we conducted a broad search of both peer‐reviewed articles and conference abstracts, it is possible that our search missed some relevant studies. Our prespecified PICO outcomes did not include PEP medication tolerability because this review was focused on PEP provision, not regimen selection, although we did look for adverse events. Our search went through October of 2023; since that date, there appear to be limited additional published studies on the PEP strategies we examined. However, one recent study of physician attitudes towards pharmacist‐prescribed PEP in the United States found general support for this approach, with greater acceptability among newer trainees compared to established physicians [37].

Future research would be useful to expand this evidence base across a range of country contexts with consideration of the diverse needs of different delivery settings and client populations. In particular, research that shows how PEP can be most effectively provided to populations who may most benefit from it, through creative outreach strategies, and with cost‐effectiveness assessments, would be helpful to inform programme decision‐making.

## 5 CONCLUSIONS

While limited, existing studies provide support for PEP in community settings and by lay health workers or through task sharing. Programmes should be expanded to include community delivery and task‐sharing to dispense, distribute, provide and monitor PEP to increase the impact of this underutilized antiretroviral HIV prevention intervention.

## COMPETING INTERESTS

The authors have no competing interests to declare.

## AUTHORS’ CONTRIBUTIONS

VM, RB, RS, H‐MAS, NF and MR conceptualized the review. CEK, PTY and RD developed the study methods and protocols, with feedback from other coauthors. CEK and RD conducted data extraction and formal analysis. CEK wrote the original draft. All authors contributed to writing and editing the review, and gave their assent to submit for publication.

## AUTHOR INFORMATION

CEK, RD and PTY are faculty members at the Johns Hopkins Bloomberg School of Public Health. VM, RB, NF and MR are staff members at the World Health Organization. H‐MAS is a staff member at UNAIDS. RS is a previous consultant for the World Health Organization and a current staff member of the Forum for Collaborative Research, University of California, Berkeley.

## FUNDING

This review was supported by the Bill and Melinda Gates Foundation (BMGF) and the United States Agency for International Development (USAID) through the World Health Organization, Department of Global HIV, Hepatitis and Sexually Transmitted Infections Programmes (WHO/HHS).

## Supporting information




**Supplementary Appendix**. GRADE evidence profiles for PICO questions

## Data Availability

The data that support the findings of this study are all publicly available through peer‐reviewed journals or conference websites.
