# The following article for this Supplement was published before the original collection was released. It can be found in its respective issue.

**DOI:** 10.1002/jia2.26527

**Published:** 2025-06-26

## Abstract

This article was intended for this Supplement issue, 28:S1, but was inadvertently published in an earlier issue of *Journal of the International AIDS Society*, issue 28:5 https://onlinelibrary.wiley.com/toc/17582652/2025/28/5. This article has also been included in the final version of *J Int AIDS Soc* issue 28:S1 as presented below for completeness. The publisher apologizes for this error and any confusion it may cause.

**When citing this article, please cite it as per its original publication in issue 28:5 as shown below**:

Kennedy CE, Dawit R, Yeh PT, Rodolph M, Ford N, Schmidt HA, et al. HIV post–exposure prophylaxis in community settings and by lay health workers or through task sharing: a systematic review of effectiveness, case studies, values and preferences, and costs. J Int AIDS Soc. 2025;28(5):e26448. https://doi.org/10.1002/jia2.26448

## Abstract 2

Kennedy CE, Dawit R, Yeh PT, Rodolph M, Ford N, Schmidt HMA, Schaefer R, Baggaley R, and Macdonald V. HIV post–exposure prophylaxis in community settings and by lay health workers or through task sharing: a systematic review of effectiveness, case studies, values and preferences, and costs. *J Int AIDS Soc*. 2025;28(5):e26448. https://doi.org/10.1002/jia2.26448

This article was intended for this Supplement issue, 28:S1, but was inadvertently published in an earlier issue of *Journal of the International AIDS Society*, issue 28:5 https://onlinelibrary.wiley.com/toc/17582652/2025/28/5. This article has also been included in the final version of *J Int AIDS Soc* issue 28:S1 as presented below for completeness. The publisher apologizes for this error and any confusion it may cause.

**When citing this article, please cite it as per its original publication in issue 28:5 as shown below**:

Kennedy CE, Dawit R, Yeh PT, Rodolph M, Ford N, Schmidt HA, et al. HIV post–exposure prophylaxis in community settings and by lay health workers or through task sharing: a systematic review of effectiveness, case studies, values and preferences, and costs. J Int AIDS Soc. 2025;28(5):e26448. https://doi.org/10.1002/jia2.26448

Kennedy CE, Dawit R, Yeh PT, Rodolph M, Ford N, Schmidt HMA, Schaefer R, Baggaley R, and Macdonald V. HIV post–exposure prophylaxis in community settings and by lay health workers or through task sharing: a systematic review of effectiveness, case studies, values and preferences, and costs. *J Int AIDS Soc*. 2025;28(5):e26448. https://doi.org/10.1002/jia2.26448

