# Supplementary material for: HIV post‐exposure prophylaxis in community settings and by lay health workers or through task sharing: a systematic review of effectiveness, case studies, values and preferences, and costs
Source: J Int AIDS Soc. 2025 May 27;28(5):e26448. doi: 10.1002/jia2.26448 (PMC12116331; doi:10.1002/jia2.26448)
Supplement: Supplementary file 1 — Supplementary Appendix. GRADE evidence profiles for PICO questions [file JIA2-28-e26448-s001.docx]

**Appendix. GRADE evidence profiles for PICO questions**

| **PICO 1: PEP offered in community settings** | | | | | | | | | | | | |
| --- | --- | --- | --- | --- | --- | --- | --- | --- | --- | --- | --- | --- |
| **Certainty assessment** | | | | | | | **№ of patients** | | **Effect** | | **Certainty** | **Importance** |
| **№ of studies** | **Study design** | **Risk of bias** | **Inconsistency** | **Indirectness** | **Imprecision** | **Other considerations** | **PEP offered by lay health workers/ task-sharing** | **no availability** | **Relative (95% CI)** | **Absolute (95% CI)** |  |  |
| **Uptake of PEP (follow-up: mean 24 weeks; assessed with: Selection of PEP compared with other biomedical prevention options (PrEP, condoms, none))** | | | | | | | | | | | | |
| 1^a^ | observational studies | serious^b^ | serious^c^ | not serious | not serious | none | The initial choice of PEP for HIV prevention was highest in the community setting (46%) compared to the OPD and ANC settings (9% and 1%, respectively). Selection of PEP remained highest in the community setting over time (23% at week 24); in the ANC and OPD settings, only 3% and 11%, respectively, ever selected PEP. | | | | ⨁◯◯◯ Very low | CRITICAL |

**ANC:** Antenatal Care; **CI:** Confidence Interval; **No:** Number; **OPD:** Outpatient Department; **PEP:** Post-Exposure Prophylaxis

a. Intervention: Dynamic choice of biomedical HIV prevention comparing community delivery vs. delivery through outpatient department (OPD) and antenatal care (ANC) settings.

b. Risk of bias: Downgraded because populations accessing the intervention through community, OPD, and ANC settings are substantially different on demographic factors (e.g., gender, age, pregnancy status).

c. Inconsistency: Downgraded for being a single study.

| **PICO 2: PEP offered by lay health workers/task-sharing** | | | | | | | | | | | | |
| --- | --- | --- | --- | --- | --- | --- | --- | --- | --- | --- | --- | --- |
| **Certainty assessment** | | | | | | | **№ of patients** | | **Effect** | | **Certainty** | **Importance** |
| **№ of studies** | **Study design** | **Risk of bias** | **Inconsistency** | **Indirectness** | **Imprecision** | **Other considerations** | **PEP offered by lay health workers/ task-sharing** | **no availability** | **Relative (95% CI)** | **Absolute (95% CI)** |  |  |
| **Uptake of PEP (follow-up: mean 24 weeks; assessed with: Selection of PEP compared with other biomedical prevention options (PrEP, condoms, none))** | | | | | | | | | | | | |
| 1^a^ | observational studies | serious^b^ | serious^c^ | not serious | not serious | none | The initial choice of PEP for HIV prevention was highest in the community setting (46%) compared to the OPD and ANC settings (9% and 1%, respectively). Selection of PEP remained highest in the community setting over time (23% at week 24); in the ANC and OPD settings, only 3% and 11%, respectively, ever selected PEP. | | | | ⨁◯◯◯ Very low | CRITICAL |
| **Uptake of PEP (assessed with: Receiving PEP prior to leaving the medical center)** | | | | | | | | | | | | |
| 1^d^ | observational studies | not serious | serious^c^ | not serious | serious^e^ | none | 16/16 (100.0%) | 5/8 (62.5%) | not estimable |  | ⨁◯◯◯ Very low | CRITICAL |
| **Completion of PEP (assessed with: Completed entire PEP course and came to follow-up appointment)** | | | | | | | | | | | | |
| 1^d^ | observational studies | not serious | serious^c^ | not serious | serious^e^ | none | 42% in intervention group vs. 32% in comparison group; n's not reported | | | | ⨁◯◯◯ Very low | CRITICAL |
| **Completion of PEP (assessed with: Presenting for follow-up within 6 months)** | | | | | | | | | | | | |
| 1^f^ | observational studies | not serious | serious^c^ | not serious | not serious | none | 19.8% (n=55) in intervention group vs. 4.3% (n=4) in comparison group; total sample size was 369 but sample size in each group not reported. | | | | ⨁◯◯◯ Very low | CRITICAL |
| **HIV acquisition (assessed with: Documented seroconversion within 6 months)** | | | | | | | | | | | | |
| 1^f^ | observational studies | not serious | serious^c^ | not serious | serious^g^ | none | 0 seroconversions in the intervention group vs. 2 in the comparison group; sample size in each group not reported. | | | | ⨁◯◯◯ Very low | CRITICAL |

**ANC:** Antenatal Care; **CI:** Confidence Interval; **No:** Number; **OPD:** Outpatient Department; **PEP:** Post-Exposure Prophylaxis

a. Intervention: Dynamic choice of biomedical HIV prevention comparing community delivery vs. delivery through outpatient department (OPD) and antenatal care (ANC) settings.

b. Risk of bias: Downgraded because populations accessing the intervention through community, OPD, and ANC settings are substantially different on demographic factors (e.g., gender, age, pregnancy status).

c. Inconsistency: Downgraded for being a single study.

d. Intervention: Adding a pharmacist to deliver PEP in an infectious disease clinic for cases referred from emergency department (ED) vs. prior to pharmacist involvement.

e. Imprecision: Downgraded for small sample size (n=24 across both arms).

f. Intervention: Pharmacist involvement in dispensing free PEP and patient education plus follow-up phone call after 3 months for sexual assault survivors in an ED vs. prior to these interventions.

g. Imprecision: Downgraded for small number of events.
